# Correlation between the Glycan Variations and Defibrinogenating Activities of Acutobin and Its Recombinant Glycoforms

**DOI:** 10.1371/journal.pone.0100354

**Published:** 2014-06-19

**Authors:** Ying-Ming Wang, Inn-Ho Tsai, Jin-Mei Chen, An-Chun Cheng, Kay-Hooi Khoo

**Affiliations:** 1 Institute of Biological Chemistry, Academia Sinica, Taipei, Taiwan; 2 Institute of Biochemical Sciences, National Taiwan University, Taipei, Taiwan; Universidad de Costa Rica, Costa Rica

## Abstract

Acutobin isolated from *Deinagkistrodon acutus* venom has been used to prevent or treat stroke in patients. This defibrinogenating serine protease is a 39 kDa glycoprotein containing terminal disialyl-capped N-glycans. After sialidase treatment, the enzyme showed similar catalytic activities toward chromogenic substrate, and cleaved the Aα chain of fibrinogen as efficiently as the native acutobin did. However, the level of fibrinogen degradation products in mice after *i.p.-*injection of desialylated-acutobin was significantly lower than the level after acutobin injection, suggesting that the disialyl moieties may improve or prolong the half-life of acutobin. Two recombinant enzymes with identical protein structures and similar amidolytic activities to those of native acutobin were expressed from HEK293T and SW1353 cells and designated as HKATB and SWATB, respectively. Mass spectrometric profiling showed that their glycans differed from those of acutobin. In contrast to acutobin, HKATB cleaved not only the Aα chain but also the Bβ and γ chains of human fibrinogens, while SWATB showed a reduced α-fibrinogenase activity. Non-denaturing deglycosylation of these proteases by peptide N-glycosidase F significantly reduced their fibrinogenolytic activities and thermal stabilities. The *in vivo* defibrinogenating effect of HKATB was inferior to that of acutobin in mice. Taken together, our results suggest that the conjugated glycans of acutobin are involved in its interaction with fibrinogen, and that the selection of cells optimally expressing efficient glycoforms and further glycosylation engineering are desirable before a recombinant product can replace the native enzyme for clinical use.

## Introduction

Viperid snake venoms usually contain serine protease isoforms with different specificities toward plasma proteins. Snake venom thrombin-like enzymes (SVTLEs) exhibit specific fibrinogenolytic activities but do not activate factor XIII, plasminogen or platelets. These enzymes generate abnormal fibrin polymers and are useful for lowering fibrinogen concentration and blood viscosity in patients [1]–[3]. For past decades, SVTLEs have been used as therapeutics to prevent or treat stroke and other cardiovascular diseases [4]–[6]. The amino acid sequences and glycan structures of SVTLE glycoproteins from different species are not similar [7] and the number and positions of their N-glycosylation sites are not conserved [8]–[10].

Most SVTLEs cleave fibrinogens either at the Aα-chain only, or at the Aα- followed by the Bβ-chain [6]. Acutobin, the major SVTLE isolated from the venom of *Deinagkistrodon acutus* (formerly named *Agkistrodon acutus*) [7], [11] is a specific α-fibrinogenase. It has been commercialized under the names of Defibrase or Acutin, and has been applied on many Chinese patients over the past three decades. Previously, we cloned the cDNA and solved the acutobin sequence [7], and showed that its complex type N-glycan structures were capped almost exclusively by terminal NeuAcα2-8NeuAc disialyl units, with the largest detectable structure consisted of as many as four such disialylated antennae [8]. Compared to ancrod [4], acutobin appears to be more effective in treating thrombosis because of its higher specific activity [12] and may have fewer adverse side effects [13]. It is unclear whether the differences in their clinical performances are due to the distinct structures of the proteins, the glycans, or both. To better understand the roles of the unusual N-glycans of acutobin, the following questions must be addressed: (a) How do the disialyl glycans affect acutobin’s functions? (b) Are the recombinant acutobins (abbreviated as ATBs) prepared from mammalian cells as good as or better than the native acutobin in terms of therapeutic efficiency? (c) How does deglycosylation by peptide N-glycosidase (PNGase F) affect the fibrinogenolytic activities, chain specificities, and stabilities of acutobin and ATBs?

In this study, we modified the acutobin glycans by sialidase and PNGase F, and studied the effects *in vitro* and *in vivo*. Additionally, we used two mammalian cell lines (HEK293T and SW1353) to prepare active ATBs which were designated as HKATB and SWATB, respectively, and analyzed their N-glycans by MALDI-TOF mass spectrometry. Besides comparing their *in vitro* fibrinogenase activities, we studied the *in vivo* effects of acutobin and ATBs on the plasma levels of fibrinogen and fibrinogen degradation products (FDP) in mice. The present study sheds light on the glycobiology of SVTLEs and should contribute toward the design and development of better defibrinogenating and antithrombotic agents.

## Materials and Methods

### Animals

Six-week-old albino mice [Bltw: CD1(ICR)] were purchased from the National Laboratory Animal Center, Taipei, Taiwan, and bred in our institutional animal facility. Permission for the animal study was obtained from the Institutional Animal Care and Use Committee (IACUC) of the National Laboratory Animal Center (Permit Number IACUC2010-096). This study was carried out in strict accordance with the recommendations in the Guide for the Care and Use of Laboratory Animals of the National Institutes of Health (USA).

### Cell Lines

Human embryonic kidney epithelial cells (HEK293T), chondrosarcoma cells (SW1353), and Chinese hamster ovary cells (CHO-K1), were obtained from American Type Culture Collection (Rockville, MD). HEK293T and SW1353 cells were cultured in Dulbecco’s modified Eagles media (DMEM) (HyClone/Thermo Scientific, USA) supplemented with 10% final concentration of fetal bovine serum (FBS) (HyClone/Thermo Scientific, USA). CHO-K1 cells were cultured in Dulbecco’s modified Eagles/F12 media (DMEM/F12) (HyClone/Thermo Scientific, USA) supplemented with 10% FBS. All the cells were cultured at 37°C in a humidified atmosphere of 5% CO_2_.

### Enzymes and Reagents

Native acutobin was purified from *D. acutus* venom (Hunan province, China) [7]. Restriction enzymes were from Promega (Madison, WI, USA). *N*-Tosyl-Gly-Pro-Arg- *p*-nitroanilide was from Sigma Aldrich (St Louis, MO, USA). Recombinant PNGase F of *Flavobacterium meningosepticnm* and *Arthrobacter ureafaciens* sialidase (neuraminidase) were purchased from Roche (Mannheim, Germany) and Bio-Rad Laboratories (Hercules, CA, USA), respectively. Pre-cast NuPAGE Novex Bis-Tris mini gels and buffers were obtained from Invitrogen Inc. (Carlsbad, CA, USA).

### Glycoprotein Modification by Sialidase and PNGase F

Removal or modification of N-glycans in acutobin and ATBs was carried out by sialidase or PNGase F treatment under mild or non-denaturing conditions such that their amidolytic activities on chromogenic substrate were not affected. Desialylation (DS-) of acutobin with 20 mU of *Arthrobacter ureafaciens* sialidase was carried out in 100 µl of 50 mM ammonium acetate (pH 6.5) at 37°C for 24 h. Deglycosylation of the enzymes by PNGase F (1.0 unit) was performed in 50 µl of 40 mM sodium phosphate (pH 7.2) at 37°C for 24 h. The molecular masses and homogeneities of the proteins, with or without the enzyme treatment, were analyzed by 4–12% NuPage SDS-PAGE.

### Vector Construction and Expression of HKATB and SWATB

The DNA sequences encoding myc epitope, His-Tag, Factor Xa cleavage site and acutobin were amplified by PCR and ligated into pSecTag2/Hygro A plasmid (Invitrogen, USA). Successful construction was verified by DNA sequencing. Three mammalian cells, HEK293T, SW1353 and CHO-K1 were used to express the ATBs. The cells were maintained in DMEM, DMEM/F12 media supplemented with glutamine, FBS and non-essential amino acids, respectively. Before transfection, HEK293T and SW1353 cells were washed at least twice with DMEM and suspended in 1 ml of DMEM, and CHO-K1 was maintained in 1 ml of DMEM/F12. About 2 µg of plasmids and 5 µl of Lipofectamine 2000 (Invitrogen, USA) were mixed and incubated at room temperature for 20 min. Then the mixture was added into the cell suspension and incubated at 37°C for 16 h. After 48 h of cultivation in fresh complete growth medium (DMEM or DMEM/F12 supplemented with 10% FBS and 200 µg/ml Hygromycin B), the cells were collected. Likewise, the control cells were transfected with blank vectors pSecTag2/Hygro A. After further cultivation for 72 h, the culture medium was harvested and the secreted proteins were analyzed by SDS–PAGE and western blotting using anti-Myc antibodies.

The culture was also scaled-up to 1 L and the secreted ATB fusion proteins were purified and concentrated using a nickel affinity column for chromatography. Factor Xa was added to the fusion proteins to a final concentration of 1% (w/w) and incubated at room temperature for 24 h to remove the tag peptide. Subsequently, the processed ATBs was injected into a Mono Q column (5/50 GL; GE Healthcare) pre-equilibrated with 50 mM Tris-HCl (pH 8.0), and then eluted with a 0–1.0 M gradient of NaCl at a flow rate of 1 ml/min. The purified ATBs were also digested by trypsin and subjected to MS/MS analysis for protein identification [14].

### Mass Spectrometry Analyses of Permethylated Glycans

The glycans were released by PNGase F from reduced, alkylated and proteases-digested acutobin, HKATB and SWATB, and then were permethylated using a modified NaOH/DMSO method before subjecting to MALDI-TOF/TOF (4700 Proteomics Analyzer, Applied Biosystems) MS mapping, as described previously [8].

### Amidase Assay using Chromogenic Substrate

Enzyme concentrations of acutobin and other derivatives were determined by absorbance at 280 nm assuming a percent extinction coefficient of 1.0 for a 1.0 mg/ml solution, and confirmed with BCA protein assay (BioRad, USA). To measure the amidolytic activities using a SpectraMax M2^e^ Microplate Reader (Molecular Devices, CA, USA), 0.5 µg of the protease was added to final volume of 0.2 ml, containing 50 µM of Tosyl-Gly-Pro-Arg- *p*-nitroanilide in 0.1 M Tris-HCl (pH 8.0), in the well of a microtiter plate (Nalge Nunc International, Rochester, NY, USA). Absorbance at 405 nm was followed immediately at 25°C and the specificity constants, *k*_cat_/*K_m_*, were calculated using GraphPad Prism. Thermal denaturation of the enzymes was studied by incubating the enzymes in 0.1 M Tris-HCl buffer for 1 h at 37°C, 55°C and 65°C. At the end of incubation, 0.5 µg of the enzymes were added to the substrate solution (50 µM) and the absorbance changes were monitored at 25°C for 20 min.

### SDS-PAGE Analyses of the Fibrinogenolytic Products

Purified acutobin, ATBs, or PNGase-treated (PNG-) enzymes (0.2 µg each) was added to 8 µg of human or rabbit fibrinogen (Sigma Aldrich, MO, USA) dissolved in 50 mM Tris-HCl (pH 8.0) in a total volume of 10 µl and incubated for different time at 37°C. The fibrinogen cleavage products were mixed with concentrated sample buffers and analyzed by SDS-PAGE. After staining and destaining, the Aα band intensity in each lane was quantified by a VersaDoc imaging system (Bio-Rad 4000MP).

### Analyses of Fibrinopeptides Released from the Clot

To detect the release of fibrinopeptide A (FpA) from fibrinogen, 1 NIH unit of α-thrombin or 0.1 µg of acutobin, HKATB, SWATB or the PNG-enzymes were added to 10 mg of human fibrinogen dissolved in 1 mL of 50 mM Tris-HCl (pH 8.0) and incubated for 1 h or 20 h at 37°C. The reactions were terminated by boiling for 5 min and the clots formed were removed by centrifuging at 12000 rpm for 15 min. The supernatants were lyophilized, re-dissolved in A buffer (0.07% trifluoroacetic acid) and subjected to reverse phase high performance liquid chromatography (RP-HPLC) using a Vydac C18 column (0.46×25 cm). A linear gradient of 10–100% of acetonitrile containing 0.07% trifluoroacetic acid was applied for elution over a period of 45 min and the absorbance at 280 nm was monitored. Peptide peaks were pooled and lyophilized. The peptides were analyzed by ESI-MS using a PE-Sciex API100 mass analyzer (Perkin-Elmer). Samples were dissolved in 20 µl of 50% CH_3_CN containing 0.1% acetic acid and injected into the analyzer for positive-mode analysis.

### Coagulation Kinetics and Clotting Time *in vitro*

Normal coagulation control plasma (ACCUCLOT Control I, Trinity Biotech, Ireland) or 2.6 mg/ml human fibrinogen in 100 µl of the buffer (25 mM Tris pH7.4/0.15 M NaCl/4 mM CaCl_2_) was kept at 37°C in a flat-bottomed microtiter plate. The enzyme in 100 µl was added (final 0.25 µg/ml) and the plate was read in the SpectraMax 340PC^384^ Microplate Reader (Molecular Devices, Sunnyvale, USA) which had been pre-warmed at 37°C. The polymerization of fibrin was measured spectrophotometrically at 405 nm every 30 seconds for 30 min [15].

Coagulation times at higher protease concentrations were also measured by an automatic coagulation analyzer (Hemostasis Analyzer KC-1; Sigma Diagnostics, St. Louis, MO, USA). Aliquots of 75 µl ACCUCLOT Control I were incubated at 37°C for 3 minutes. After incubation, 75 µl of the protease stock solution was added to trigger coagulation. Pre-warmed thrombin solution (3 NIH U/ml) was used as control.

### Euglobulin Clot Lysis Assay

The assay was performed using a modified version of the method described by Smith *et al*. [16]. Euglobulin fraction was prepared by adding 350 µl of normal coagulation control plasma (Trinity Biotech., St. Louis, USA) to 6.3 ml of working acetic acid prepared by adding 1.5 ml of 1% acetic acid to 90 ml reagent grade H_2_O. The solution was placed on ice for 20 minutes and then centrifuged at 3500 rpm for 15 min. The supernatant was discarded and the pellet was resuspended in 700 µl of a solution containing 150 mM NaCl and 2.6 mM sodium borate, pH 9.0 and kept at 37°C. Acutobin or the indicated proteases were then added to 150 µl of the resuspended euglobulin fraction in a microplate. Finally 150 µl of 25 mM CaCl_2_ was added to initiate clotting. The plate was then placed in SpectraMax M2^e^ Microplate Reader (Molecular Devices, CA, USA) that had been pre-warmed at 37**°**C, and the absorbance was measured at 340 nm for 10 h.

### Plasma Fibrinogen and FDP Levels in Mice

Experimental mice were kept in a 12/12 h light/dark cycle with food and water *ad libitum*. To determine the plasma fibrinogen concentration, venous blood was collected from the mice in 3.8% sodium citrate (9∶1 v/v) and centrifuged at 2500 *g* for 15 minutes. The test solutions were prepared by diluting plasma 10-fold with a buffer containing 30 mM imidazole, 125 mM sodium chloride and 0.1% sodium azide (pH 7.35). 100 µl of the diluted plasma was then mixed with thrombin (33.3 U/ml) and the clotting time was measured [17]. The plasma fibrinogen concentration was then determined by intrapolating or extrapolating from a calibration curve prepared by mixing serially-diluted plasma (0.1∼2.4 mg/ml) with thrombin.

Acutobin or DS-acutobin (in 100 µl of PBS) was injected intraperitoneally (*i.p.*) into male ICR mice (6 weeks old weighing 28–30 g). Control mice were similarly injected with PBS. Blood was extracted from the jugular vein of the animals at 1, 2, 3, 4 or 24 h after enzyme injection and anticoagulated with 3.8% sodium citrate (9∶1 v/v). FDP concentrations were determined using NANOPIA P-FDP kit (Daiichi Pure Chemicals Co Ltd., Tokyo, Japan) according to the manufacturer’s protocol. Two way analysis of variance (ANOVA) with repeated measures followed by Bonferroni’s post-hoc analysis was used for statistical comparisons. In another experiment, FDP concentrations were determined 4 h after the injection (*i.v.*) of acutobin, HKATB, SWATB or their PNG-treated forms. One way ANOVA followed by Bonferroni’s post-hoc analysis was employed in this case. GraphPad Prism software was used for statistical analyses. *P* values <0.05 are considered to be statistically significant.

## Results

### The *in vitro* and *in vivo* Effects of DS-acutobin

**Figure 1:**
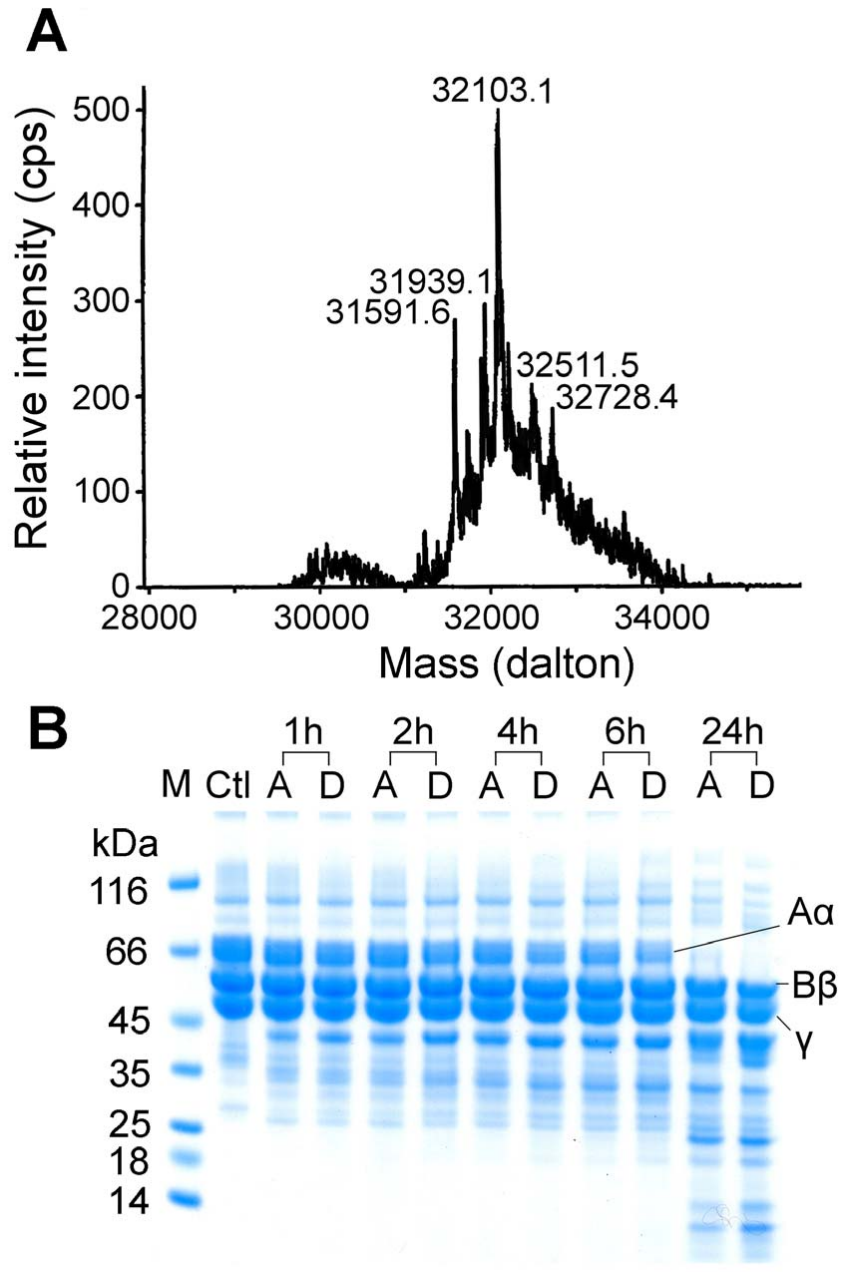
Characterization of DS-acutobin. (A) Analysis of DS-acutobin mass by ESI-MS spectrometry. (B) Comparison of human fibrinogen hydrolyses by 20 µg/ml of acutobin and DS-acutobin. The products were analyzed by SDS-PAGE after 1∼24 h. Abbreviations used: A, acutobin; D, DS-acutobin; ctl, control with fibrinogen only.

Acutobin was incubated with sialidase for 24 h under a mild condition such that its enzyme activity remained intact (Table 1). The ESI-MS results of DS-acutobin revealed a mixture of glycoproteins distributed at around 32103 Da (Fig. 1A), whereas the masses of native acutobin was distributed at around 36476 Da [8]. The results suggest that most of the sialyl groups, or an average of about 15 sialyl per acutobin molecule, were removed by sialidase. DS-acutobin showed similar catalytic activity toward chromogenic substrate as the native acutobin (Table 1). Fibrinogen is a hexamer containing two sets of three different chains, namely Aα, Bβ, and γ; both acutobin and DS-acutobin at 20 µg/ml cleaved the Aα chains with similar efficacy (Fig. 1B). Additionally, 0.3 µg DS-acutobin clotted the ACCUCLOT solution in 12 seconds, similar to the coagulation time elicited by 0.3 µg of the native acutobin. These results suggest that although the removal of the terminal disialyl caps of the glycans reduced the surface negative charges, it probably did not affect the catalytic center conformation of acutobin.

**Table 1:** Catalytic efficiencies of acutobin, DS-acutobin, ATBs, and PNG-enzymes toward chromogenic substrate Tosyl-Gly-Pro-Arg-*p*-nitroanilide.

Protease	10^5^ × *k*_cat_/*K*_m_ (M^−1^S^−1^)
acutobin	1.3±0.1
DS-acutobin	1.4±0.2
PNG-acutobin	1.6±0.2
HKATB	1.3±0.3
PNG-HEKATB	1.3±0.3
SWATB	1.1±0.3
PNG-SWATB	1.0±0.2

**Figure 2:**
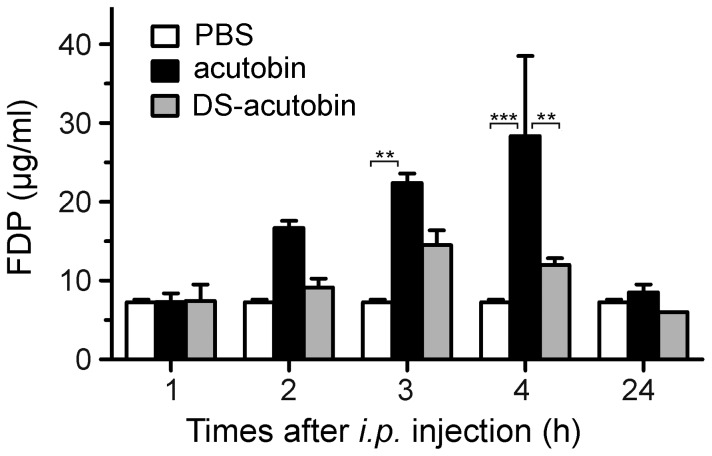
Comparison of the activities of acutobin and DS-acutobin *in vivo*. ICR mice were *i. p.* injected with 250 ng/g body weight of acutobin or DS-acutobin. Blood was collected at different time points, and the plasma FDP levels were determined. Values shown are means ± SEM (n = 3). ** indicates *P*<0.01, *** indicates *P*<0.001.

To evaluate the contribution of disialylated glycans for the *in vivo* defibrinogenase activity, we compared the plasma levels of FDP in ICR mice after *i.p.* injection of acutobin or DS-acutobin. Blood was extracted from the mice at different time points up to 24 h after the enzyme injection, and the FDP concentrations were determined (Fig. 2). Notably, the FDP level in mice injected with 250 ng/g acutobin increased rapidly in the first 3∼4 h, while the FDP level of DS-acutobin injected mice increased slowly and declined after 3∼4 h. Compared to the control mice, acutobin increased the mouse FDP level by 3∼5-fold. Apparently, acutobin hydrolyzed fibrinogen/fibrin faster than DS-acutobin in mice. After injected with high doses, some acutobin-treated mice showed temporary discomfort and decreased mobility, but DS-acutobin-treated mice showed no signs of illness throughout the experiment.

### Purification and Fibrinogenase Specificities of ATBs

**Figure 3:**
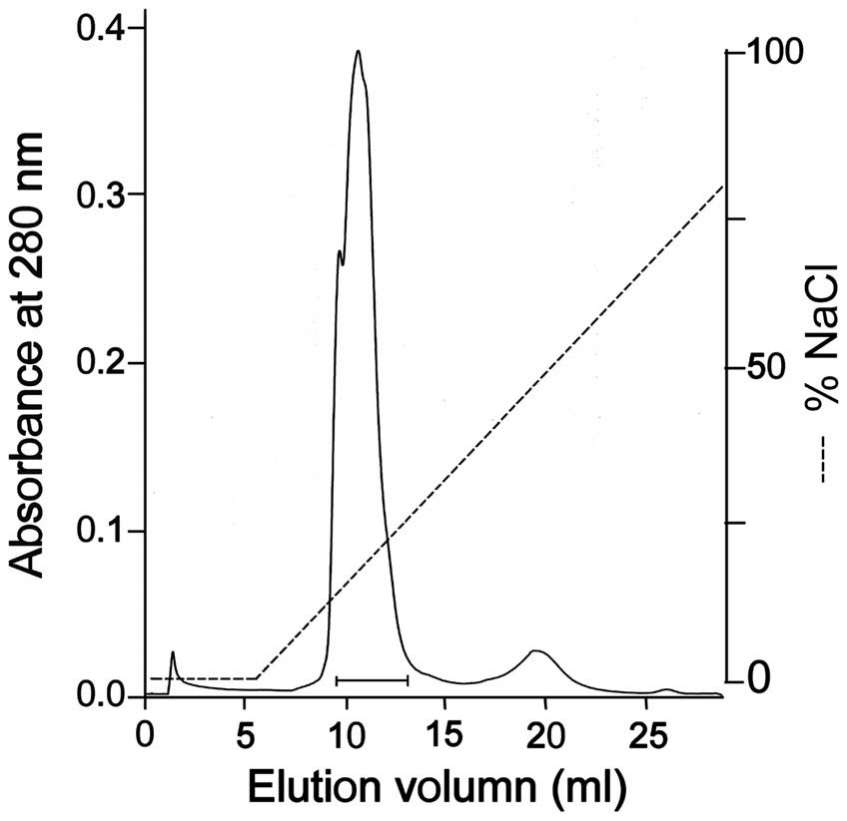
Purification of the recombinant acutobin by Mono Q column. After eluted from Ni-affinity columns and removal of the fusion peptides by Factor Xa, HKATB or SWATB was loaded into a Mono Q column (5/50 GL) that had been pre-equilibrated with 50 mM Tris-HCl buffer (pH8.0), and the enzymes were eluted with a NaCl gradient from 0∼1.0 M. The fractions containing HKATB (indicated by a bar) were pooled. The purification of SWATB showed similar results.

**Figure 4:**
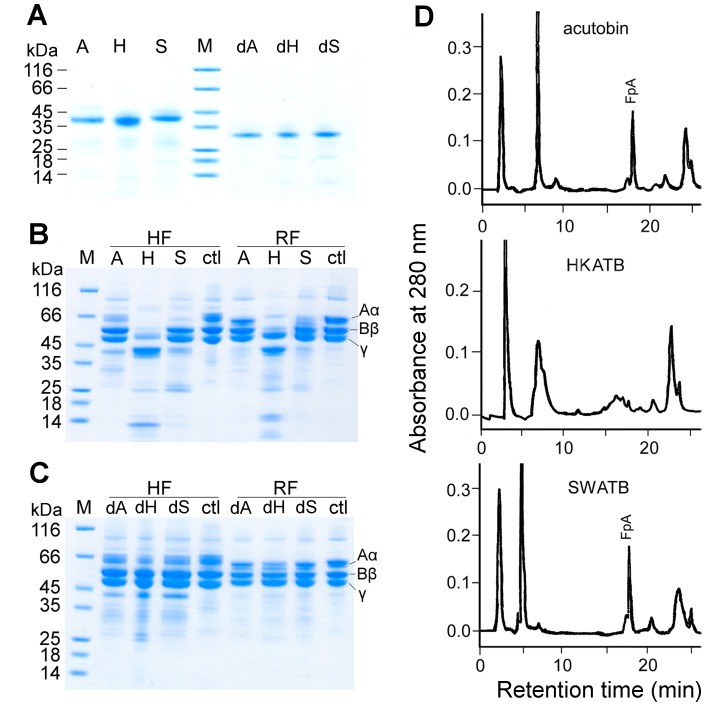
Hydrolysis of human and rabbit fibrinogens by acutobin and its glycoform variants. (A) Purified acutobin, HKATB, SWATB and PNGase-treated enzymes were analyzed by SDS-PAGE. (B) Hydrolyses of human fibrinogen (HF) or rabbit fibrinogen (RF) by acutobin and ATBs. Enzyme concentrations used were 20 µg/ml. (C) Hydrolysis of the two fibrinogens by PNGase-treated enzymes. Abbreviations A, H, S, dA, dH, and dS: acutobin, HKATB, SWATB, and their PNGase-deglycosylated forms, respectively; M, molecular weight markers; ctl, control with fibrinogen only. (D) HPLC isolation and identification of FpA in the supernatants above the clot generated by acutobin, HKATB and SWATB, respectively.

Recombinant fusion proteins of acutobin were successfully harvested from both HEK293T, SW1353 cell cultures and purified. After Factor Xa processing and purification by ion exchanger chromatography (Fig. 3), the yields of HKATB and SWATB were found to be 0.6 and 0.4 mg/L, respectively. However, the yield of the fusion protein from CHO-K1 cells was too low to be purified. Both purified HKATB and SWATB appeared as single bands on polyacrylamide gel (Fig. 4A) and had molecular masses very similar to that of acutobin (38 kDa). The peptide MS/MS analysis of HKATB revealed 58% sequence coverage when Mascot search was performed and the coverage rose to 86% if the glycoproteins were pre-treated with PNGase F before the trypsin hydrolysis step (Table 2).

**Table 2:** Peptide mass fingerprinting analysis of PNG-HKATB.

Determ., Da	Calcul., Da	Score	Modification	Positions	Amino acid sequence
500.26	1497.79	10	1 SCMC^a^	1–13	VIGGVECDINEHR
433.69	865.36	25		62–68	FDDEQGR
731.84	1461.67	92		62–73	FDDEQGREPIEK
777.34	1552.66	86	1 SCMC^a^, 1 NQ^b^	74–85	YFYNCSNNLTTR
502.28	1002.55	57		86–93	DKDIMLIR
757.64	3026.57	55	1 SCMC^a^	94–121	LDRPV….PSVGSVCR
757.90	3027.55	113	1 SCMC^a^, 1 NQ^b^	94–121	LDRPV….PSVGSVCR
580.79	1159.58	69		122–132	VMGWGAISPSR
1224.59	2447.16	152	2 SCMC^a^	133–153	DVLPD….NAECR
868.76	2603.26	115	2 SCMC^a^	133–154	DVLPD….NAECRR
429.92	1286.72	28		154–164	RAYPRLPATSR
377.88	1130.62	28		155–164	AYPRLPATSR
869.88	1737.76	98	2 SCMC^a^	165–180	TLCAGVMQGGIDSCNR
870.38	1738.74	38	2 SCMC^a^, 1 NQ^b^	165–180	TLCAGVMQGGIDSCNR
877.89	1753.75	79	2 SCMC^a^, 1MO^c^	165–180	TLCAGVMQGGIDSCNR
1216.90	3647.66	119	2 SCMC^a^	181–214	DSGGPL….PALYTK
1222.23	3663.65	116	2 SCMC^a^, 1MO^c^	181–214	DSGGPL….PALYTK
622.29	1242.57	54		215–223	VYDYNDWIR
1243.07	2484.14	2	1 SCMC^a^	215–236	VYDYN….TAACPP
631.29	1260.57	40	1 SCMC^a^, 1 NQ^b^	224–236	SITAGNTTAACPP

The deglycosylated HKATB was reduced and alkylated by iodoacetamide before trypsin hydrolysis. Masses determined for the resultant peptides were match with those calculated based on the amino acid sequence of acutobin.

a SCMC: S-carbamidomethyl Cys.

b NQ: asparagine deamidation.

c MO: methionine oxidation.

Using purified fibrinogen (8 µg) and 0.2 µg enzyme in 10 µl of Tris-HCl buffer at 37°C for 2 h, the specificities of acutobin, HKATB and SWATB for human and rabbit fibrinogens were compared. The product analyses by SDS-PAGE revealed that acutobin hydrolyzed rabbit fibrinogen much slower than human fibrinogen; this is in agreement with an earlier finding on the acutobin purified from Taiwanese *D. acutus* venom [11]. Notably, acutobin and SWATB cleaved only the Aα chain of human and rabbit fibrinogens but HKATB hydrolyzed the Aα, Bβ and γ chains of both fibrinogens under the same condition (Fig. 4B). The results also confirmed that HKATB cleaved the rabbit fibrinogen subunits much faster than acutobin, and SWATB also had a slightly higher activity than acutobin toward the rabbit Aα-chains. Thus, glycan alterations in the ATBs may affect either their specificity or reactivity toward fibrinogen, the native substrate of acutobin.

### Effects of PNGase Treatment on the Enzymes Activities and Specificities

To investigate the functional importance of the multivalent glycosylation, a non-denaturing process was carried out to remove the majority of the N-glycans from acutobin and ATBs. Treatment of acutobin and ATBs with PNGase F for 24 h yielded sharp bands of lower molecular weights on the SDS-PAGE gel (Fig. 4A). MALDI-TOF mass analysis of PNG-acutobin revealed a molecular mass of 29.5 kDa (not shown), which is slightly higher than the predicted mass of 26 kDa for the totally deglycosylated acutobin [7]. Since there is no evidence of O-glycosylation in acutobin and the masses of acutobin N-glycans are around 2.7–4.9 kDa [8], the PNGase treatment probably removed an average of three glycans from each acutobin or ATB molecule. Similar partial resistance to PNGase F hydrolysis was also observed in the cases of other venom serine proteases, including Russelobin [5] and BJ-48 [18].

As shown in Table 1, the amidolytic activities of PNG-enzymes were similar to those of the untreated enzymes toward Tosyl-Gly-Pro-Arg-*p*-nitroanilide. But all the PNG-enzymes hydrolyzed only the Aα chains of human fibrinogen, and the rates of hydrolysis were also lower than those of the original enzymes (Fig. 4C vs. Fig. 4B). Notably, PNG-HKATB hydrolyzed the human or rabbit fibrinogen much slower than HKATB and could no longer hydrolyze the Bβ and γ chains (Fig. 4C).

### HPLC and Mass Analyses of Fibrinopeptides

The fibrinopeptide A (FpA) released from the fibrin clots formed by acutobin, ATBs or their PNG-treated counterparts was analyzed by RP-HPLC (Fig. 4D). The released FpA showed a mass of 1536.6 Da corresponding to the N-terminal 16 residues of the Aα chain. We found that acutobin and PNG-acutobin released FpA within 1 h of incubation, and peaks corresponding to fibrionopeptide B were not detected. In contrast, the HPLC profile of the HKATB products showed fragments that were eluted before FpA, but both HKATB and PNG-HKATB could not release FpA even after 20 h. Interestingly, FpA was detected in the clot formed by SWATB after 20 h, supporting the notion that SWATB can cleave the fibrinogen Aα chain but at a much slower rate.

### N-glycan Structures of HKATB and SWATB

**Figure 5:**
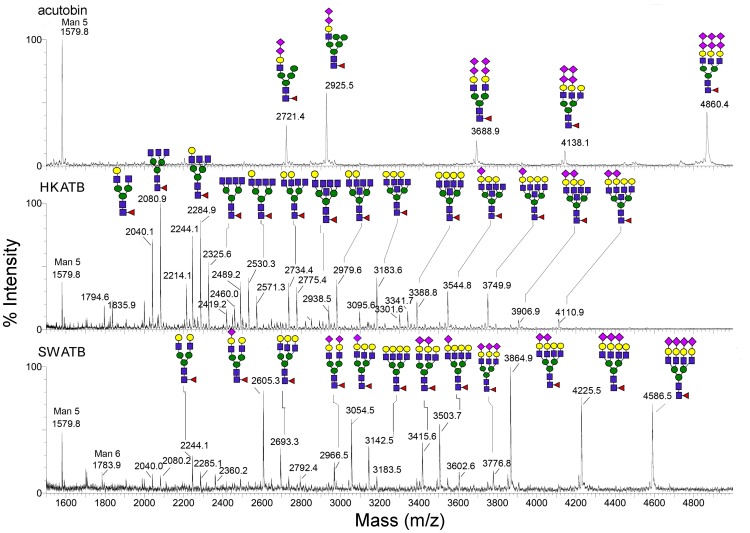
MALDI-MS mapping of the permethylated N-glycans released from acutobin, HKATB and SWATB. Glycosyl compositions were assigned based on the detected m/z values of the [M+Na]^+^ molecular ion signals and the annotated sugar structures were deduced based on further biosynthesis considerations. Only the detailed structures for the acutobin glycans have been reported [8]. The symbols used for annotation of the assigned peak conform to the standard representation recommended by the Consortium for Functional Glycomics. In MS terms, circle represents hexose, square for N-acetyl-hexosamine, triangle for fucose and diamond for sialic acid.

The N-glycans were released from HKATB and SWATB and profiled by MALDI-MS (Fig. 5). As expected, different cells produced different glycoforms of ATBs and a higher degree of N-glycosylation heterogeneity was observed on both ATBs relative to that on native acutobin. In general, they all carried a range of bi-, tri and tetra-antennary complex type structures with varying degrees of sialylation (Fig. 5). N-glycans from HKATB alone comprise many species carrying additional HexNAc attributable to incompletely galactosylated and/or bisecting GlcNAc, whereas most antennary GlcNAc in SWATB were apparently galactosylated and further sialylated to different extent. Neither of the two ATBs carried N-glycans capped by the unique terminal disialyl units as found in the N-glycans from native acutobin. Due to the heterogeneity of the sugar chains in the glycoproteins, ESI-mass spectra of intact acutobin, HKATB and SWATB showed clusters of signals centered around 36476, 34210 and 39310 Da, respectively (not shown). After subtracting the theoretical mass (26105 Da) of their polypeptide chain [7], their average mass increments contributed by N-glycosylation are 10371, 8105 and 13205 Da, respectively. Thus, the averaged N-glycan masses of HKATB are lower than those of acutobin and SWATB. This conclusion is consistent with the MALDI-MS profile afforded by the N-glycans released from their respective enzymes, showing that the major glycans carried on HKATB and SWATB have fewer and more antennae than acutobin, respectively (Fig. 5).

### Effect of Deglycosylation on Thermostability of the Enzymes

**Figure 6:**
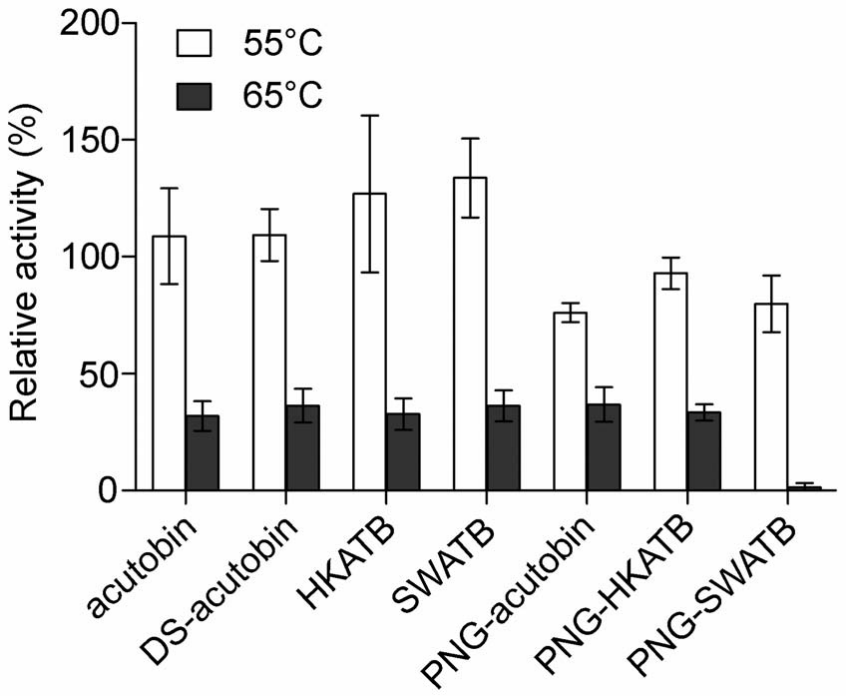
Thermal stability of different glycoforms of acutobin. The indicated enzymes were incubated at 37°C, 55°C and 65°C for 1 h. The hydrolytic activities toward Tosyl-Gly-Pro-Arg-*p*-nitroanilide were assayed at 25°C and an enzyme concentration of 2.5 µg/ml. The remaining activity of each enzyme after incubation at 37°C was taken as 100%.

Serine proteases are known to be thermal stable at temperatures below 50°C in neutral buffer. To assess the contribution of glycosylation to their thermal stability, acutobin, ATBs and their PNGase-treated counterparts were separately incubated at 37°C, 55°C and 65°C for 1 h, respectively. The remaining amidolytic activities were then assayed at 25°C. We found that all proteases were stable at 37°C. Unlike native acutobin and ATBs which remained stable at 55°C, the deglycosylated enzymes showed 25–30% inactivation after incubation at 55°C. Incubation at 65°C for 1 h resulted in 63–68% inactivation for most of these enzymes, and PNG-SWATB was almost completely inactivated (Fig. 6). Thus, the glycans of acutobin and HKATB could protect the enzymes against thermal denaturation while SWATB was less stable probably because of the structure or nature of its glycan moieties.

### Coagulation Kinetics of Acutobin and Other Glycoforms

**Figure 7:**
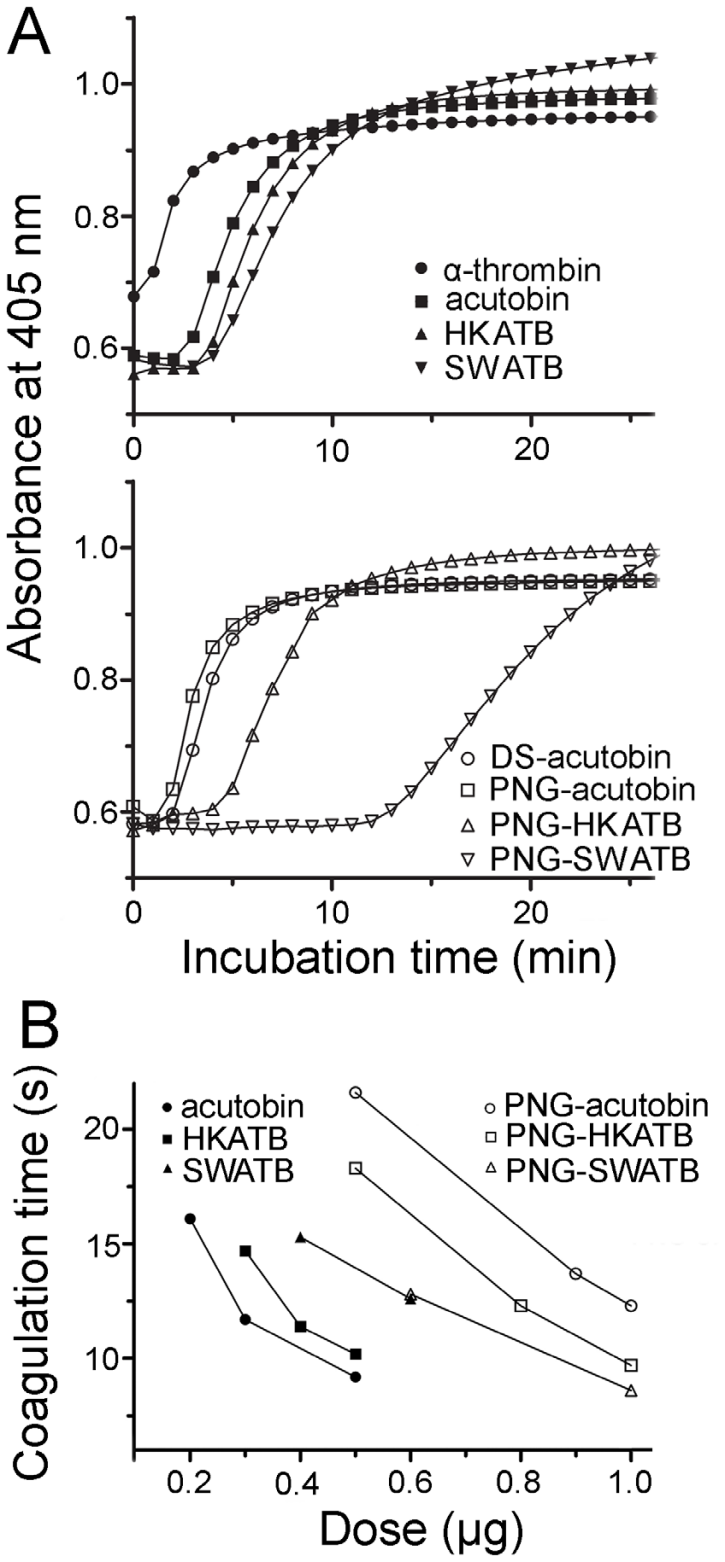
Coagulation of human plasma by acutobin and its various glycoforms. (A) Coagulation of human plasma was monitored spectrophotometrically at 37°C after addition of the indicated enzymes (final 0.25 µg/ml). The curves are averaged results of three experiments. (B) Coagulation times of human plasma in the presence of various amounts of the enzymes in a total volume of 150 µl were determined by a coagulation analyzer. Data shown are based on the averaged results of two or three experiments.

The tertiary and quaternary structural changes of fibrin/fibrinogen molecules during hydrolysis and polymerization could be monitored spectrophotometrically. As shown in Fig. 7A, human plasma was coagulated by 1 NIH unit of α-thrombin (as a positive control) or 0.25 µg/ml (final concentration) of acutobin or other enzymes, and these enzymes elicited an increase in absorbance at 405 nm within 12 min. The polymerization rates followed the order of acutobin/PNG-acutobin>HKATB/PNG-HKATB>SWATB/PNG-SWATB (Fig. 7A). This *in vitro* assay also revealed that at the same enzyme concentration, both DS-acutobin and PNG-acutobin exhibit higher fibrinogenolytic activities than PNG-HKATB and PNG-SWATB. Additionally, using purified human fibrinogen at a physiological concentration of 2.6 mg/ml, we found a similar order of the polymerization rate, *i.e.* acutobin>HKATB>SWATB>PNG-ATBs (data not shown). Remarkably, the polymerization rates were faster in the fibrinogen solution than in human plasma, suggesting that the activities of ATBs could be suppressed by some plasma components.

The measurement of plasma coagulation time has been a standard protocol for quality control in the manufacture of acutobin as therapeutics. Fig. 7B shows the results of coagulation time measured on a coagulation analyzer and using enzyme concentrations higher than those used in Fig. 7A. To achieve the same clotting time of 12 seconds, the required amounts of acutobin, HKATB, and SWATB under the assay conditions were 0.30, 0.38 and 0.66 µg (in 150 µl), while those of PNG-acutobin, PNG-HKATB, and PNG-SWATB were 1.1, 0.81 and 0.68 µg, respectively. Thus, partial (about 75%) deglycosylation significantly reduced the clotting rates of acutobin and HKATB, reflecting the contribution of the glycans to the clotting abilities of both fibrinogenases, although PNG-SWATB showed similar low clotting rate as SWATB. Our results also suggest that the clotting time assay (Fig. 7B) is more sensitive than the polymerization assay (Fig. 7A) to reflect the effects of glycan variations.

### Results of Euglobulin Clot Lysis Assay

**Figure 8:**
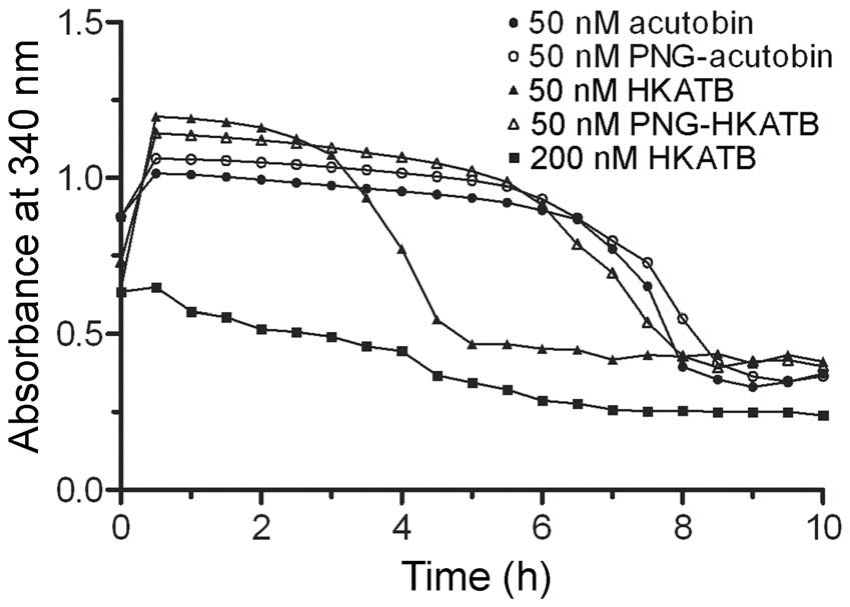
Euglobulin-clot-lysis assay. The stabilities of clots formed by the indicated enzymes were compared based on the results of this assay. The curves are averaged results of two experiments.

Euglobulin clot lysis assay was performed to compare the lysis rate of the clots formed. Like the thrombin control (1U), clots formed by 50 nM acutobin or PNG-acutobin lysed between 6–10 h (Fig. 8). Similar results were obtained with clots induced by SWATB and PNG-SWATB (data not shown). However, the clot formed by 50 nM HKATB lysed significantly faster than the clots formed by other proteases, and when the HKATB concentration was increased to 200 nM, no clot was formed. The results could be explained by the non-specific cleavage of fibrinogen by HKATB, consistent with the HPLC results of the fibrinogen cleavage product (Fig. 4D). Notably, deglycosylation by PNGase F corrected the abnormality of HKATB in the euglobulin clot lysis assay.

### Plasma Fibrinogen and FDP Level in Mice After Injected the Enzyme

**Figure 9:**
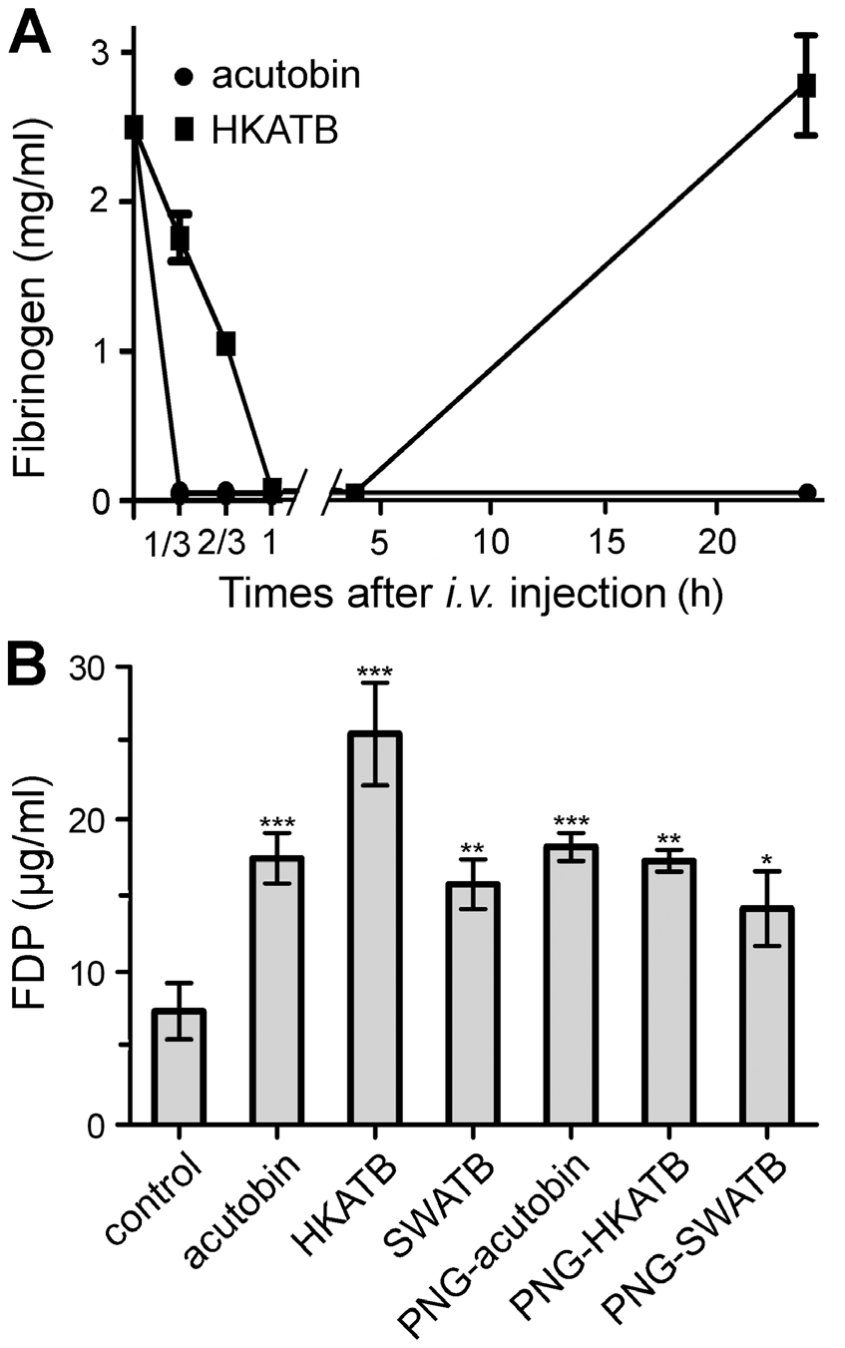
Comparison of the plasma fibrinogen and FDP levels in vivo. (A) ICR mice were *i.v.* injected with 5 ng/g body weight of acutobin or HKATB and the plasma fibrinogen concentrations were determined. (B) The indicated enzymes (50 ng/g body weight) were *i.v.* injected into ICR mice. After 4 h, the plasma FDP levels were determined and compared with those of the control mice (injected PBS only). Values shown are means ± SEM (n = 3). * indicates *P*<0.05, ** indicates *P*<0.01, *** indicates *P*<0.001.

In an early report, rabbit blood became incoagulable within 1–4 h when the rabbit was *i.v.* injected with 50 ng/g body weight of acutobin [11]. In our study, when 50 ng/g of acutobin or HKATB was *i.v.* injected into ICR mice, the mouse fibrinogen level dropped rapidly and was too low to be measured within 1 h. When a lower injected dose (5 ng/g) of acutobin or HKATB was injected, the decline of plasma fibrinogen could be measured. We found that the plasma fibrinogen level in HKATB-injected mice decreased at a slower rate compared to acutobin-injected mice (Fig. 9A). After 24 h, the fibrinogen level in HKATB-injected mice was replenished but the fibrinogen level in acutobin-injected mice remained undetectable. Thus, acutobin is a more potent and robust defibrinogenating agent compared to HKATB *in vivo*.

Elevation of FDP level has been frequently observed in the blood of pitviper envenomed victims, indicating systemic fibrinogenolysis and fibrinolysis [19]. We compared the FDP concentrations in mice 4 h after *i.v.* injection of acutobin and other enzymes. The plasma FDP levels of all the mice increased after the animals were injected with 50 ng/g body weight of the enzymes, relative to the control (Fig. 9B). Notably, the FDP increases after acutobin and HKATB injection were significantly higher than those after PNG-acutobin and PNG-HKATB injection, confirming the contribution of the glycans to the *in vivo* defibrinogenating effects. Relative to HKATB, SWATB elicited only a small increase in plasma FDP levels (as did PNG-SWATB), especially when injected via the *i.p.* route.

## Discussion

N-glycosylation often contributes to proper protein folding and secretion during its biosynthesis in the Golgi and endoplasmic reticulum [20]. The N-glycans of Russelobin, and the sialyl groups of batroxobin were found to play a role in protecting the enzymes against neutralization by physiologically relevant inhibitors such as α_2_-macroglobulin [5]. Our results showed that the removal of three out of the four N-glycans from acutobin and ATBs decreased their fibrinogenolytic activities and stability at elevated temperatures. The thermal protectant effects of N-glycans have also been observed in many other SVTLEs [18], [21], [22]. The lower stability of SWATB relative to acutobin and HKATB nevertheless suggests that glycans with increased size or branching (Fig. 5) may destabilize acutobin. It is likely that different glycan structures may confer differential thermal protection or other physiological effects of a glycoprotein.

Most of the four N-glycosylation sites of acutobin carry the disialylated glycans, and the full range of 1 to 4 disialyl LacNAc antenna [8] is distinct from the sugars in ancrod and batroxobin [9], [10]. It has been well known that sialylation of otherwise exposed termini may extend the circulatory half-life of many serum glycoproteins by reducing their clearance through hepatic asialoglycoprotein receptor [23]. Chemically linking polysialic acids to proteins was shown to improve the half-life of the protein without adversely affecting their function [24], [25]. Results in Fig. 2 and Fig. 9 support the notion that the disialyl-capped N-glycans may increase or improve the *in vivo* half-life of acutobin. The mechanism possibly involves the binding interaction of acutobin with certain immune cells carrying specific siglecs that may preferably bind the disialyl epitopes [26]. Such pharmacokinetic advantage would not be observed for DS-acutobin (Fig. 2) and HKATB (Fig. 9A), which do not contain similarly disialylated N-glycans. Notably, the removal of sialyl residues from acutobin (Fig. 1B) and other SVTLEs (e.g. bilineobin [27] and okinaxobin II [28]) did not affect their specificities or reactivities toward fibrinogen *in vitro*. The sialyl or disialyl units of these SVTLEs therefore probably do not play a direct role in the interactions with fibrinogen.

Previous preparations of venom serine proteases from *Escherichia coli* inclusion bodies suffered either from improper folding [29], or from poor clotting activities [30], [31]. Recombinant SVTLEs with “high mannose type” of N-glycans have been prepared from *Pichia* yeast [32], [33], but their specificities and pharmacological activities were not investigated. Current glycoprotein therapeutics are usually produced by particular human or mammalian cells, and their N-glycans vary according to the activities of glycosyltransferases in their ER-Golgi systems. CHO cells can express more extensively sialylated glycoproteins with molecular weights higher than those produced by HEK293, and in some cases may retard the secretion of the recombinants [34]. The present study found that ATB fusion proteins were not secreted from CHO-K1 cells that could be attributed to the unfavorable glycosylation in the cells.

We have, for the first time, expressed recombinant venom fibrinogenases with different N-glycans from animal cells, while conserving the protein sequences and the glycosylation sites (Fig. 5). HKATB cleaved the Aα, the Bβ, and the γ chains of human and rabbit fibrinogen (Fig. 4B), but its cleavage sites on human fibrinogen became less specific and the enzyme did not release FpA (Fig. 4D). Moreover, HKATB clotted human plasma slower than acutobin did (Fig. 7B), and was less effective than acutobin in reducing fibrinogen levels in mice (Fig. 9A). SWATB showed lower stability and lower activities for human fibrinogen. The N-glycans of SWATB possibly are too bulky than is optimal for the enzyme to bind and cleave human fibrinogen. Thus, the fibrinogen specificities and enzymatic activities of ATBs could be affected by the size and branching of their glycan structures, the safety and clinical efficacy of using ATBs in place of native acutobin are questionable at present.

The amino acid sequence identities among SVTLEs are about 60% or higher, but their sequence identities to thrombin (with one N-glycosylation site) are less than 30% [27], [28], [35], [36]. Most SVTLEs are fibrinogenases and do not show multiple biological activities as thrombin does, and their clotting efficacies are much lower than thrombin [21], [35]. Moreover, each SVTLE could show rather different chain-selectivities or preferences for fibrinogens from different species [11], [36]. Previous attempts to correlate the amino acid sequences of SVTLEs with their fibrinogen-chain specificities had only limited success [7], [35]–[37]. It was postulated that the charge distribution profiles together with the topographic variations around the catalytic interface define the selectivity of SVTLEs for various plasma proteins [7], [38]. The interaction of SVTLEs with macromolecular substrates is certainly not only dependent on their catalytic sites. Notably, most of the SVTLEs are highly glycosylated relative to other venom enzymes. This may imply certain associations between fibrinogen and the glycans of a SVTLE during the venom evolution. Thrombin possesses charged exosites and so possibly also the venom enzymes. The contribution of sugars to the interactions between a glycoprotein enzyme and its substrate could be achieved by specific structural component of the glycans or the sugar clustering effects [39]. Acutobin and the SVTLEs from *Bothrops pauloensis* and *Trimeresurus elegans* [22], [36] showed reduced fibrinogenolytic activities (Fig. 4C) and reduced clotting activities (Fig. 7B) after PNGase treatment. However, after PNGase treatment, the human FpA generation rate of bilineobin and *Bothrops* protease A was increased [27], [40]. Both bilineobin and protease A, with 6 and 8 potential N-glycosylation sites, respectively, appear to be hyper-glycosylated, the reduction of their glycans possibly facilitated the hydrolysis of α-fibrinogen *in vitro*. Taken together, the *N*-glycans of SVTLEs possibly participate in the interactions with fibrinogen, as also postulated by the results of the crystallographic studies on SVTLEs [41], [42].

Acutobin contains four glycosylation sites at N77, N81, N100 and N231 (the acutobin numbering [8]); among them only the N231 is conserved among most of the SVTLEs which release only FpA [9], [10], [27]. The role of each N-glycan site in acutobin remains to be explored by site directed mutagenesis. Another serine protease isoform Da-36 has been isolated from *D. acutus* venom from Central-Western provinces of China. The amino acid sequence of Da-36 is 63% identical to that of acutobin and contains only two potential glycosylation sites at N57 and N100 (acutobin numbering system) [21]. Da-36 can hydrolyze all the three subunits of fibrinogen. Acutobin, ancrod, batroxobin and many other SVTLEs [35] released only FpA from fibrinogens, but AhV TL-I [41], chitribrisin [31], bilineobin [27], okinaxobin II [28], *Lachesis muta muta* SVTLEs [35], and Russelobin [5] released both FpA and FpB from fibrinogens. The structural determinants for the fibrinogen specificities of these SVTLEs remain elusive.

Fibrinogen depleting agents may help to remove the blood clot blocking the artery and re-establish blood flow to the affected areas of the brain after an ischemic stroke [4], [13]. The acutobin therapeutics contain about 5 µg (or 5–10 Units) per vial [43], and the effective doses used (about 5–15 Units) to treat Chinese patients are around 0.1 µg/kg body weight, or 0.01–0.03 nM in the blood of patients after *i.v.* injection. Our study revealed that acutobin and HKATB at 2–5 ng/g body weight could efficiently reduce the fibrinogen level *in vivo*. In contrast, much higher concentrations of acutobin or ATBs were used *in vitro* to demonstrate similar effects in human plasma. Whether and how the glycans in acutobin and other SVTLEs may facilitate their interactions with fibrinogen under the *in vivo* vascular circulation condition remains to be clarified.

## Conclusion

Knowledge of the structure-function relationships of SVTLE glycoproteins is essential to advance our understanding of their reaction mechanisms and potential clinical use. Using acutobin as a model molecule, we have confirmed that the multivalent glycans of SVTLEs are important not only for maintaining structural stability but also for substrate specificity and possibly pharmacokinetics. We also showed that the disialyl-caping of acutobin is beneficial for the *in viv*o function of this therapeutic enzyme. HEK293T cells can be used to prepare reactive ATBs with good yields. However, *in vitro* coagulation and fibrinogenolysis assays and *in vivo* studies revealed that the clinical efficacy and specificity of HKATB may not be as good as that of native acutobin. Apparently, the specificities of SVTLE for fibrinogens from different mammalian species could be affected by the locations, numbers, and structures of their glycans. Thus, optimization of the expression cell lines and the enzyme protein engineering are required before recombinant SVTLEs become suitable for clinical applications.
